# Revising the Freshwater *Thelohania* to *Astathelohania* gen. et comb. nov., and Description of Two New Species

**DOI:** 10.3390/microorganisms10030636

**Published:** 2022-03-17

**Authors:** Cheyenne E. Stratton, Lindsey S. Reisinger, Donald C. Behringer, Jamie Bojko

**Affiliations:** 1 Fisheries and Aquatic Sciences, University of Florida, Gainesville, FL 32653, USA; c.stratton@ufl.edu (C.E.S.); lreisinger1@ufl.edu (L.S.R.); behringer@ufl.edu (D.C.B.); 2 Emerging Pathogens Institute, University of Florida, Gainesville, FL 32611, USA; 3 School of Health and Life Sciences, Teesside University, Middlesbrough TS1 3BA, UK; 4 National Horizons Centre, Teesside University, Darlington DL1 1HG, UK

**Keywords:** Microsporidia, crayfish, disease ecology, parasite, taxonomy, *Thelohania*

## Abstract

Crayfish are common hosts of microsporidian parasites, prominently from the genus *Thelohania*. *Thelohania* is a polyphyletic genus, with multiple genetically distinct lineages found from freshwater and marine environments. Researchers have been calling for a revision of this group for over a decade. We provide evidence that crayfish-infecting freshwater *Thelohania* are genetically and phylogenetically distinct from the marine *Thelohania* (Clade V/Glugeida), whilst also describing two new species that give further support to the taxonomic revision. We propose that the freshwater *Thelohania* should be transferred to their own genus, *Astathelohania* gen. et comb. nov., in a new family (Astathelohaniidae n. fam.). This results in the revision of *Thelohania contejeani* (*Astathelohania contejeani*), *Thelohania montirivulorum* (*Astathelohania montirivulorum*), and *Thelohania parastaci* (*Astathelohania parastaci*). We also describe two novel muscle-infecting *Astathelohania* species, *A. virili* n. sp. and *A. rusti* n. sp., from North American crayfishes (*Faxonius* sp.). We used histological, molecular, and ultrastructural data to formally describe the novel isolates. Our data suggest that the *Astathelohania* are genetically distinct from other known microsporidian genera, outside any described family, and that their SSU rRNA gene sequence diversity follows their host species and native geographic location. The range of this genus currently includes North America, Europe, and Australia.

## 1. Introduction

Microsporidia are intracellular, spore-forming parasites that commonly infect animals in freshwater environments [1]. In one common group of freshwater arthropods, crayfish, microsporidiosis is often referred to as “cotton-tail” or “porcelain disease”, since the muscle tissue of infected individuals often turns opaque white [2]. Infections are usually chronic and result in muscle function loss and ultimately death [2]. Several microsporidian genera have been identified from crayfish globally, including: *Cambaraspora*, *Nosema*, *Ovipleistophora*, *Pleistophora*, *Thelohania*, and *Vavraia* [3,4,5,6]. Four of these genera (*Cambaraspora*, *Ovipleistophora*, *Pleistophora*, *Thelohania*) infect North American crayfish species; however, the presence of only two genera (*Ovipleistophora* and *Cambaraspora*) has been confirmed using molecular tools [5,6]. One genus, the *Thelohania* (Thelohaniidae; Clade V), is polyphyletic and genetically distinct between marine and freshwater environments—only freshwater *Thelohania* infect crayfish [7].

The *Thelohania* are one major group of crayfish pathogens. To date, three species have been formally identified (*Thelohania contejeani*, *Thelohania montirivulorum*, *Thelohania parastaci*) [8,9,10]. *Thelohania contejeani* was the first crayfish-infecting *Thelohania* species to be described and has three known hosts in Europe [11,12,13,14,15]. Two *Thelohania* species have been described from Australia, *T. montirivulorum* and *T. parastaci*, which were identified from common yabby (*Cherax destructor*) [9,10]. All three of these species share a similar development with a dimorphic pattern of sporogony, and free binucleate spores and uninucleate spores contained within sporophorous vesicles (SPVs). In North America, there have been two suspected *T. contejeani* infections in crayfish. *Thelohania contejeani* was reported in 1979 in virile crayfish (*Faxonius virilis*) in Ontario, Canada, using morphology (spore measurements) [16,17]. The same parasite was also reported in a signal crayfish (*Pacifastacus leniusculus*) from California in 1983, based on spore morphology [18]. *Pacifastacus leniusculus* has been diagnosed with *T. contejeani* in its invasive range in Europe, confirmed using molecular diagnostics [14]. An unofficial *Thelohania*, *T. cambari*, was described from Appalachian brook crayfish (*Cambarus bartonii*) in Georgia and South Carolina based on morphology [19]. *Thelohania cambari* has not been reported since its initial description in 1950 and there are no molecular or ultrastructural data available. In recent years, a high diversity of crayfish-infecting microsporidia has been reported from North America with supporting DNA sequence data, but the taxonomy surrounding historic, morphology-based observations is unreliable, and it is unknown whether any were truly *Thelohania* species [5,6,20].

Historically, the genus *Thelohania* (Thelohaniidae) was considered to house over 80 described species from terrestrial, freshwater, and marine environments. The genus had a broad geographical and host range that included vertebrates, crustaceans, and terrestrial insects [21]. There are no genetic or ultrastructural data available for the *Thelohania* type species *T. giardia*, a parasite of *Crangon crangon* (marine decapod shrimp) [8,21]. The description of the genus was broad, leading to many microsporidia being incorrectly classified into the *Thelohania* [8,22]. The first ‘true’ *Thelohania* with gene sequence data available, *T. butleri*, was identified from Canadian pink shrimp (*Pandalus jordani*) off the coast of British Columbia, Canada [21]. *Thelohania butleri* is considered a ‘true’ *Thelohania* because it infects a marine decapod host and has a similar development to *T. giardia*, phylogenetically grouping within the Thelohaniidae family and Clade V of the Microsporidia [8,21]. The availability of genetic data for a ‘true’ *Thelohania* has already led to the taxonomic revision of two terrestrial *Thelohania* species [23,24]. The genetic data provided by this ‘true’ *Thelohania* member suggest the placement of freshwater, crayfish-infecting microsporidia in this genus is phylogenetically inaccurate, despite possible morphological similarities. Genomic data for *T. contejeani* also support that it is not a Clade V (Glugeida) or Thelohaniidae member [25]. Genetically, the crayfish-infecting, freshwater *Thelohania* currently reside within an ‘orphan’ lineage (also termed Clade VI), including *Hamiltosporidium*, *Neoflabelliforma*, and *Areospora* [26,27,28,29,30]. Therefore, the genetically distinct freshwater *Thelohania* genus requires taxonomic revision at both the genus, family, and possibly higher taxonomic levels.

Here we propose a taxonomic revision, removing the freshwater *Thelohania* from this genus and associated family (Thelohaniidae), and erecting a new genus and family *Astathelohania* n. gen. (Astathelohaniidae n. fam.) to represent the phylogenetically-grouping, freshwater, crayfish-infecting, microsporidia that share high levels of genetic similarity to one another, but not the marine *Thelohania*. We describe two new species of *Astathelohania* n. gen., *Astathelohania virili* n. sp. and *Astathelohania rusti* n. sp., which infect *F. virilis* and rusty crayfish (*Faxonius rusticus*), respectively. These novel isolates are the first confirmed cases of freshwater *Thelohania* (now *Astathelohania*) infections in North America based on a combination of histological, molecular, and ultrastructural data.

## 2. Materials and Methods

### 2.1. Crayfish Locality and Collection

Four *F. virilis* adults, some presenting white muscle tissue, were collected from two lakes in Wisconsin, USA (Table 1). Animals were stored in lake water and immediately brought back to Trout Lake Station where they were dissected. In addition, two *F. rusticus* presenting white muscle tissue were collected from their native range in Ohio, USA (Table 1). These individuals were shipped overnight to the Fisheries and Aquatic Sciences laboratory at the University of Florida where they were dissected for histopathological analysis.

### 2.2. Histopathology

For histopathological screening, crayfish were dissected to obtain antennal gland, eye, gill, gonad, gut, heart, hepatopancreas, muscle, and nerve tissue. These tissues were preserved in Davidson’s Freshwater Fixative (35.5% tap water, 31% 95%-ethanol, 22% formaldehyde, 11.5% glacial acetic acid) for 24–48 h and then moved to 70% ethanol. The tissues were wax-embedded, sectioned (3–4 μm), mounted on glass slides, and stained with hematoxylin and alcoholic eosin as specified in Bojko et al. [5]. Histology slides were screened using a Leica DM500 microscope. Biopsies of the antennal gland, gill, hepatopancreas, and muscle tissue were also fixed in 96% molecular grade ethanol for molecular diagnostics and a third biopsy of the same tissues placed into 2.5% glutaraldehyde in a 0.1% sodium cacodylate buffer for transmission electron microscopy (TEM).

### 2.3. Transmission Electron Microscopy

Microsporidia-infected muscle tissue was transferred from 2.5% glutaraldehyde in a 0.1% sodium cacodylate buffer to 4% paraformaldehyde with 2.5% glutaraldehyde in 0.1 M sodium cacodylate (pH 7.24). A Pelco BioWave Pro laboratory microwave (Ted Pella, Redding, CA, USA) aided with processing of fixed tissues. Samples were washed in 0.1 M sodium cacodylate (pH 7.24) then postfixed in 2% osmium tetroxide followed by two water washes. Samples were dehydrated in a graded ethanol series (25% to 100% in 5–10% increments) followed by 100% acetone. The samples were resin infiltrated using a ARALDITE/Embed epoxy resin and Z6040 embedding primer (Electron Microscopy Services (EMS), Hatfield, PA, USA) in increments of 3:1, 1:1, 1:3 anhydrous acetone:ARALDITE/Embed followed by 100% ARALDITE/Embed.

Resin infiltrated samples were cured for 72 h at 60 °C before semi-thick sections (500 nm) were stained with toluidine blue. Ultra-thin sections were collected on carbon coated Formvar 100 mesh grid (EMS, Hatfield, PA, USA). Sections were stained with 2% aqueous uranyl acetate and lead citrate (EMS, Hatfield, PA, USA). Sections were viewed with an FEI Teenai G2 Spirit Twin TEM (FEI Corp., Hillsboro, OR, USA) and digital images were captured with a Gatan UltraScan 2k × 2k camera and Digital Micrograph software (Gatan Inc., Pleasanton, CA, USA). All morphology measurements were acquired from TEM images and ImageJ software [31].

### 2.4. Molecular Diagnostics

Microsporidia-infected muscle tissue underwent DNA extraction using Qiagen’s DNeasy Blood and Tissue kit (Qiagen, Hilden, Germany) following the manufacturer’s protocol. Extracted DNA was used in a Promega ‘Flexi-Tag’ PCR (4Promega, Madison, WI, USA) consisting of 2.5 mM MgCl_2_, 1 mM dNTPs, 0.25 μL Promega Taq polymerase, 10 μL buffer, 1 μM forward primer V1F (5′-CACCAGGTTGATTCTGCCTGAC-3′), 1 μM reverse primer MC3r (5′-GATAACGACGGGCGGTGTGTACAA-3′) in a 50 μL reaction volume [32]. The thermocycler conditions for the reaction consisted of an initial denature at 94 °C for five minutes followed by 35 cycles of 94 °C–55 °C–72 °C, with each temperature held for one minute, and a final extension period at 72 °C for seven minutes. The resulting amplicons were visualized using gel electrophoresis on a 1.5% agarose gel. The microsporidia-specific amplicon size was ~1100 bp. The bands were excised from the gel and extracted using Qiagen’s gel extraction kit (Qiagen, Hilden, Germany). The amplicons were sent for sequencing using Eurofins Genomics (eurofinsgenomics.com; accessed on 20 January 2022) for both forward and reverse orientation.

### 2.5. Phylogenetics and Genetic Comparisons

A maximum-likelihood (ML) phylogenetic tree was constructed for representative species from across the Microsporidia (*n* = 150), including all available *Thelohania* isolates and those sequenced in this study. Sequences were downloaded from NCBI, or provided by authors, and aligned using MAFFT in CIPRES [33], resulting in 2519 bp comparable columns (including gaps). The alignment was uploaded to the IQtree server [34] for ML tree construction, resulting in a tree inferred from 1000 bootstrap replicates and based on the evolutionary model: GTR+F+I+G4, according to Bayesian information criterion (BIC). The resulting tree was annotated in FigTree v.1.4.4. (tree.bio.ed.ac.uk/software/figtree/; accessed on 22 January 2022) and rooted to a *Metchnikovella* isolate.

The sequence demarcation tool v.1.2. [35] was used to compare the genetic similarity of the rRNA (SSU) gene for all available freshwater *Thelohania* isolates, along with *T. butleri* (marine; Glugeida), other genera in the ‘orphan lineage’ (*Hamiltosporidium*, *Neoflabelliforma*, *Areospora*), and the new isolates sequenced in this study.

Additional phylogenetic comparison was conducted for crayfish, using a 742 bp fragment of the mitochondrial cytochrome oxidase 1 gene, representing four families: Cambaridae (*n* = 23), Cambaroididae (*n* = 3), Astacidae (*n* = 5), Parastacidae (*n* = 23). The ML phylogenetic analysis was conducted in IQtree [34], after alignment in CLC genomics workbench v.22 (MUSCLE), using 1000 bootstraps and evolutionary model TIM2+F+I+G4 (according to BIC).

## 3. Results

### 3.1. Pathology, Ultrastructure, and Development for Microsporidiosis in Faxonius virilis

**Figure 1:**
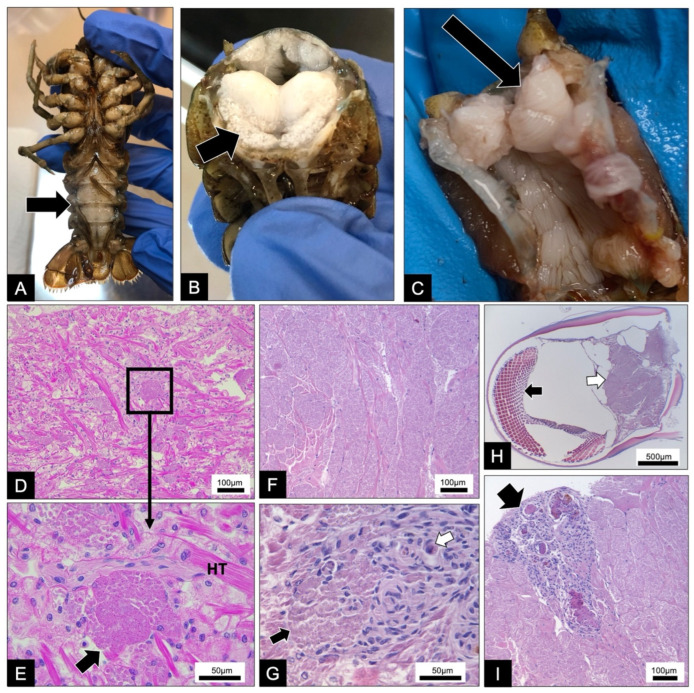
Gross pathology and histopathology of microsporidian infections in *Faxonius virilis* and *Faxonius rusticus*: (**A**) muscle tissue of infected crayfish is white and visible through the ventral cuticle of the abdomen (black arrow); (**B**) a transverse section of the abdomen reveals white muscle tissue (black arrow) presumably due to infection; (**C**) during dissection, white muscle tissue throughout the body cavity was white (black arrow) from the infection; (**D**) heart tissue with groups of developing spores; (**E**) a higher magnification of (**D**) of one cluster of developing spores in the heart tissue (HT) and the evident sporophorous vesicles containing the spores (black arrow); (**F**) abdominal muscle tissue exhibiting a heavy microsporidian infection; (**G**) high magnification image of a cluster of spores (black arrow) developing within the heart tissue and the production of granulomas (white arrow); (**H**) microsporidian spores (white arrow) developing within the muscle stalk of the eye (black arrow); (**I**) an immune response to the microsporidian infection in the abdominal muscle resulting in the production of several granulomas (black arrow).

One of the four *F. virilis* specimens exhibited signs of microsporidiosis in the form of white muscle tissue, visible through the ventral cuticle of the abdomen (Figure 1A–C). This individual had a loss of righting response and subsequent decline in physiological condition in captivity. The remaining three *F. virilis* individuals did not exhibit clear signs of gross pathology. Histological screening of all individuals revealed microsporidian spores developing within sporophorous vesicles (SPV) within the sarcolemma of host skeletal and heart muscle fibers (Figure 1D–I). Multiple developmental stages were observed during histological screening.

**Figure 2:**
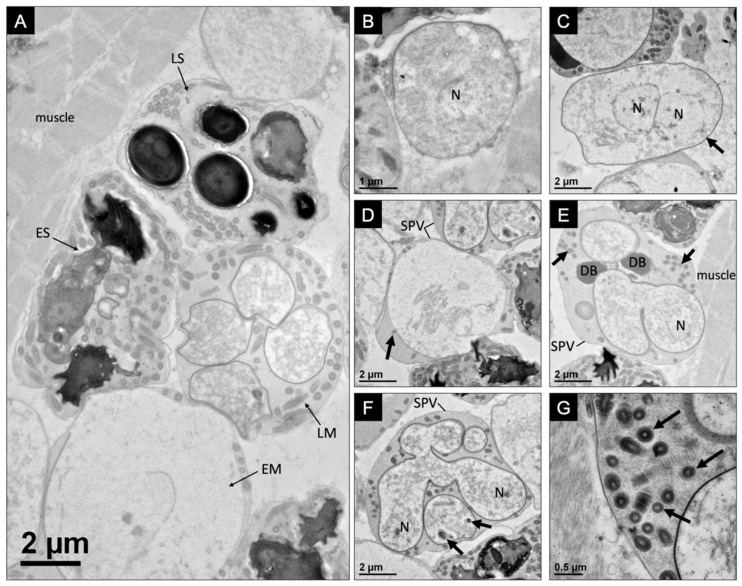
Merogony of *Astathelohania virili* n. sp. (**A**) This image identifies an early merogonal stage (EM), a late merogonal stage (LM), an early sporoblast stage (ES), and a late sporoblast stage (LS). Each stage is developing within their own sporophorous vesicle in close proximity to one another and near host muscle tissue; (**B**) a binucleate meront (N = nucleus) with a thin cell wall; (**C**) a binucleate meront (N = nuclei) with a thickening cell wall (arrow); (**D**) sporophorous vesicle (SPV) developing from meront with tubular-like structures present (arrow); (**E**) division of sporont into sporoblasts within SPV with one visible nucleus (N). SPV contains dense bodies (DB) and tubular-like structures (arrows); (**F**) another dividing sporont within an SPV. Electron-dense organelles beginning to develop within developing sporonts (arrows); (**G**) high magnification image of tubular-like structures (arrows) found within late merogony SPVs.

**Figure 3:**
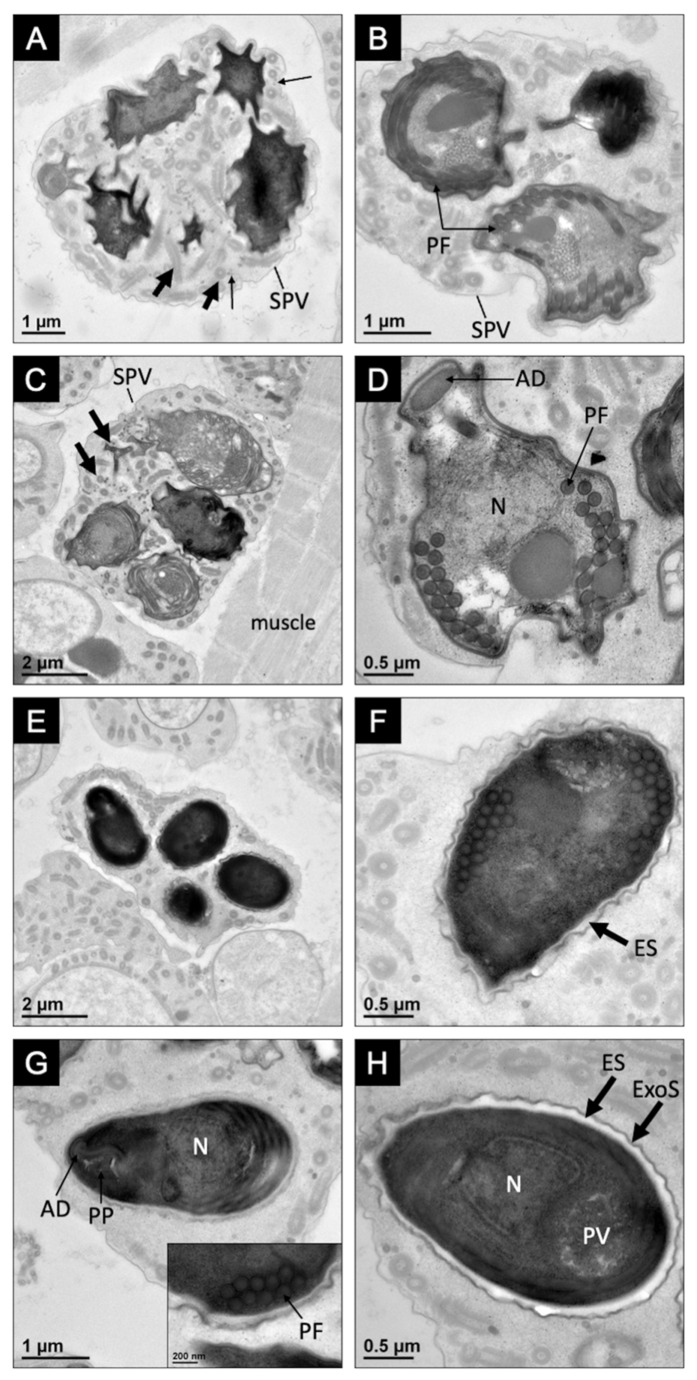
Sporogony and spore ultrastructure of *Astathelohania virili* n. sp. (**A**) Sporoblasts developing within a single sporophorous vesicle (SPV) that contains microtubular-like (small arrow) and tubular-like structures (large arrow); (**B**) sporoblasts beginning to develop electron-dense organelles including the polar filament (PF); (**C**) sporoblasts continuing to develop within SPV containing tubular-like structures (arrows) in close association with host muscle tissue; (**D**) a uninucleate (N) sporoblast with developing organelles including the anchoring disc (AD) and polar filament (PF); (**E**) near mature spores within an SPV; (**F**) spore with thickening endospore (ES); (**G**) uninucleate spore with a well-developed anchoring disc (AD) and bilaminar polarplast (PP). Inset shows fine details of polar filament and the two-layer arrangement; (**H**) a uninucleate spore with posterior vacuole and spore wall consisting of thickening electron-lucent endospore (ES) and electron-dense exospore (ExoS).

The developmental pattern for the microsporidium-infecting *F. virilis* occurred within the sarcolemma of the muscle fibers, with various stages of spore development occurring within close proximity to one another (Figure 2A). The development began with a binucleate meront in direct contact with the host cytoplasm and often proximally associated with host muscle fibers (Figure 2B,C). SPVs (8.1 ± 0.7 μm in diameter; *n* = 10, SD) were observed to house developing meronts, which divided into up to eight early sporonts (Figure 2D). During sporogony, a sporogonial plasmodium, which is presumably formed from the merging of binucleate counterparts and subsequent meiosis (not observed), divides into up to eight uninucleate sporoblasts via rosette-like division (Figure 2E,F). Dense bodies created by aggregations of granules were observed within the SPVs in the early stages of sporogony prior to the formation of individual sporoblasts (Figure 2E). As the sporonts developed into sporoblasts, electron-dense organelles began to develop (Figure 2F). Microtubular-like (73 ± 10 nm in diameter; *n* = 10, SD) and tubular-like (241 ± 26 nm in diameter; *n* = 10, SD) structures were observable within the episporontal space, which became more numerous as the development of the sporoblasts progressed (Figure 2G and Figure 3A). Sporoblasts were characterized by a thick electron-dense plasmalemma and the early development of the organelles, including the polar filament and anchoring disc (Figure 3B–D).

All mature spores observed were uninucleate. Uninucleate mature spores were contained within SPVs and were oval in shape, with a wider posterior end (Figure 3E). Mature spores were 3.4 ± 0.1 μm (*n* = 7, SD) in length and 2.0 ± 0.3 μm (*n* = 10, SD) in width, with 16–17 coils of the polar filament (118 ± 3 nm in diameter; *n* = 10, SD) arranged in two or three layers (Figure 3F). The mature spore ultrastructure included an anchoring disc, bilaminar polarplast, a coiled polar filament, and a posterior vacuole (Figure 3G). The spore wall was composed of an electron-lucent endospore (82 ± 12 nm; *n* = 10, SD) and an electron-dense exospore (25 ± 3 nm; *n* = 10, SD), which thinned at the apex of the spore above the anchoring disc (Figure 3H).

### 3.2. Pathology, Ultrastructure, and Development for Microsporidiosis in Faxonius rusticus

Two *F. rusticus* specimens exhibited signs of microsporidiosis, with white muscle tissue visible through the ventral cuticle of the abdomen. Upon dissection, white musculature was seen throughout the body cavity of the specimen (Figure 1A–C). Histological screening of the infected *F. rusticus* individuals revealed microsporidian spores developing within SPVs within the sarcolemma of the hosts’ skeletal and heart muscle fibers (Figure 1D–I). Multiple developmental stages were observed during our histological screening, which were observed in greater detail using TEM.

**Figure 4:**
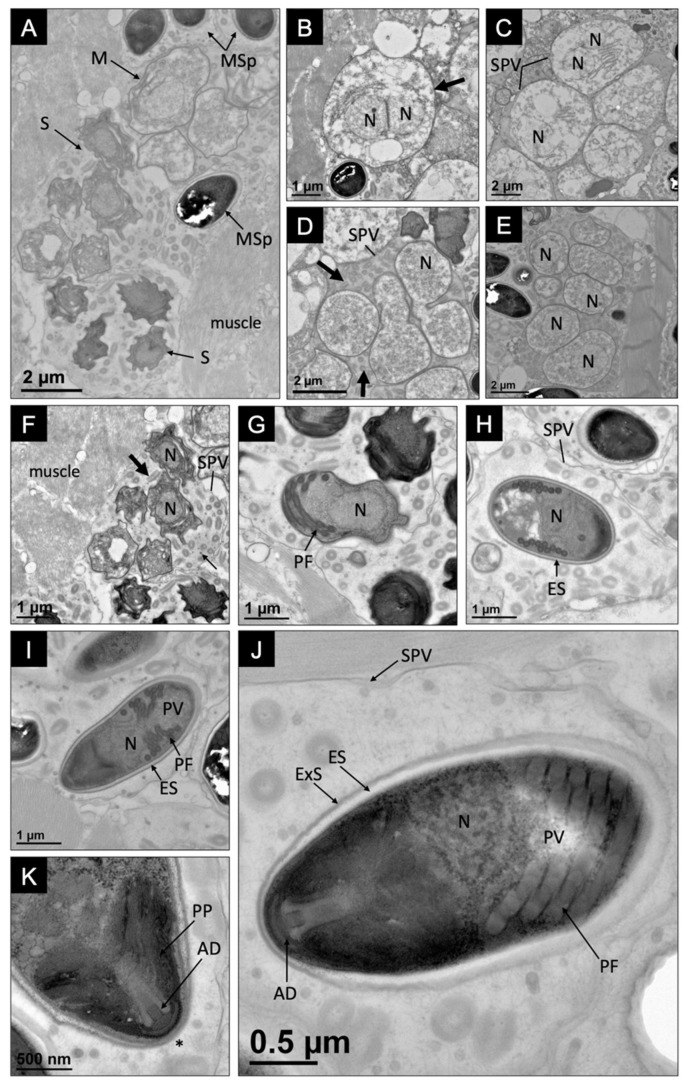
Intracellular developmental cycle of *Astathelohania rusti* n. sp. within the muscle tissue of *Faxonius rusticus*. (**A**) This image identifies a merogony stage (M), a sporogony (S), and mature spores (MSp) developing within their own sporophorous vesicles (SPV) in close proximity to host muscle tissue; (**B**) a binucleate meront (N = nuclei) with a thickening cell wall (arrow); (**C**) nuclei dividing within meronts and SPVs developing around each meront; (**D**) division of sporont within SPV. SPV contains tubular-like structures (arrows); (**E**) early uninucleate (N) sporoblasts maturing within SPVs; (**F**) uninucleate sporoblast developing within SPV with both microtubular-like (small arrow) and tubular-like structures (large arrow) present; (**G**) uninucleate sporoblast developing organelles including the polar filament (PF); (**H**) a near mature uninucleate spore developing within an SPV with a thickening electron-lucent endospore (ES); (**I**) a near mature uninucleate spore with a thicker endospore and well-developed polar filament (PF) and posterior vacuole (PV); (**J**) the ultrastructure of a mature uninucleate spore developing within an SPV includes a posterior vacuole (PV), polar filament (PF), anchoring disc (AD), and a spore wall with a thick electron-lucent endospore (ES) and electron-dense exospore (ExoS); (**K**) shows the fine details of the bilaminar polarplast (PP) and anchoring disc (AD) with the spore wall thinning above the anchoring disc (*).

The development of the novel microsporidium occurred within the sarcolemma of the host muscle fibers and various developmental stages were visible in close proximity to one another within individual SPVs (Figure 4A). Mature spores were not found to be dimorphic, and all observed spores were uninucleate. The development of this microsporidium began with large binucleate meronts developing in direct contact with host cytoplasm (Figure 4B). Meronts were not contained within an SPV and had a simple plasmalemma.

Merogony included the development of an SPV (5.2 ± 0.6 μm in diameter; *n* = 10, SD), which appeared to develop from the plasmalemma (Figure 4C). The binucleate meront progressed into a rosette-shaped plasmodium, which divided to form eight uninucleate sporoblasts (Figure 4D,E). Microtubular-like (70 ± 9 nm in diameter; *n* = 10, SD) and tubular-like structures (244 ± 32 nm in diameter; *n* = 10, SD) were abundant within the episporontal space at this stage of development. As the sporoblasts continued to develop, their plasmalemma thickened and became more electron dense. They developed organelles, beginning with the polar filament (Figure 4F,G). As the sporoblast progressed into a mature spore, a thick, electron-lucent endospore became apparent (Figure 4H).

The ultrastructure of a mature spore included an anchoring disc, a bilaminar polarplast, a posterior vacuole, and a polar filament, which coiled 13–14 times (141 ± 14 nm in diameter; *n* = 10, SD) (Figure 4I–K). The mature spores were uninucleate and oval, with a wider posterior end. The spores were 3.2 ± 0.5 um (*n* = 10, SD) in length and 1.7 ± 0.3 um (*n* = 10, SD) in width with a spore wall composed of an electron-lucent endospore (57 ± 18 nm; *n* = 10, SD) and electron-dense exospore (25 ± 6 nm; *n* = 10, SD), which thinned at the apex of the spore above the anchoring disc (Figure 4J,K).

Table 2 provides morphological information for the two new species, and provides a comparison to other related species, following a table provided by Moodie et al. [9].

### 3.3. Genetic Similarity and Phylogenetic Placement of the Novel Microsporidians

**Figure 5:**
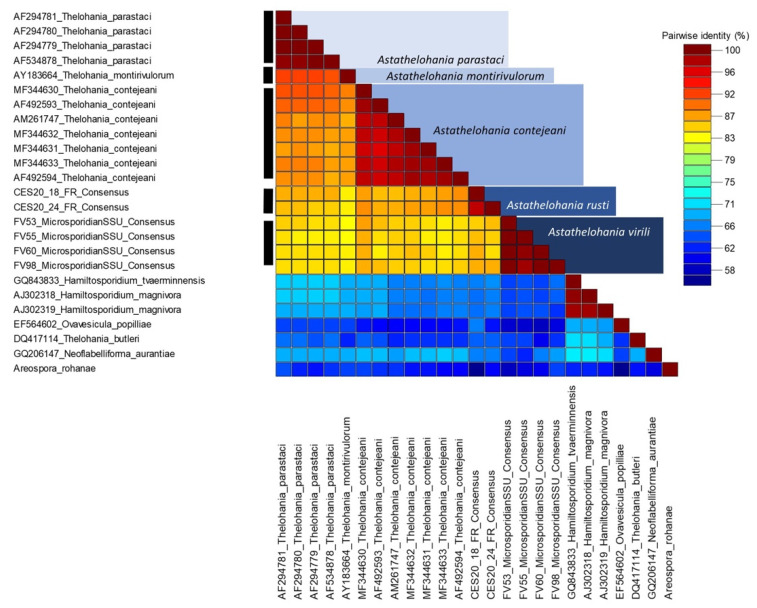
A similarity matrix reflecting the percent similarity between different *Astathelohania* (=*Thelohania*) rRNA (SSU) gene isolates. The key provides a color scheme that reflects the similarity between isolates (blue/low to red/high). The figure was designed using the sequence demarcation Tool v1.2 [35].

The four microsporidian SSU sequence isolates from *F. virilis* were identical to one another, as were the two isolates from *F. rusticus* (Figure 5); however, the novel isolates from each host were genetically distinct (98% coverage; 83.71% similarity; e-value: 0.0). A 775 bp sequence from the novel microsporidium-infecting *F. virilis* (OM630068) showed 84.79% similarity to a *T. contejeani* isolate (MF344630: 97% coverage; e-value: 0.0) from *Austropotamobius pallipes* in Italy. Similarly, a 735 bp sequence from the novel microsporidium-infecting *F. rusticus* (OM630067) was 87.36% similar to the same *T. contejeani* isolate (MF344630: 96% coverage; e-value: 0.0). Our sequence demarcation plot highlights the genetically distinct freshwater *Thelohania* species based on the geographic location from which the isolates were found (Figure 5).

**Figure 6:**
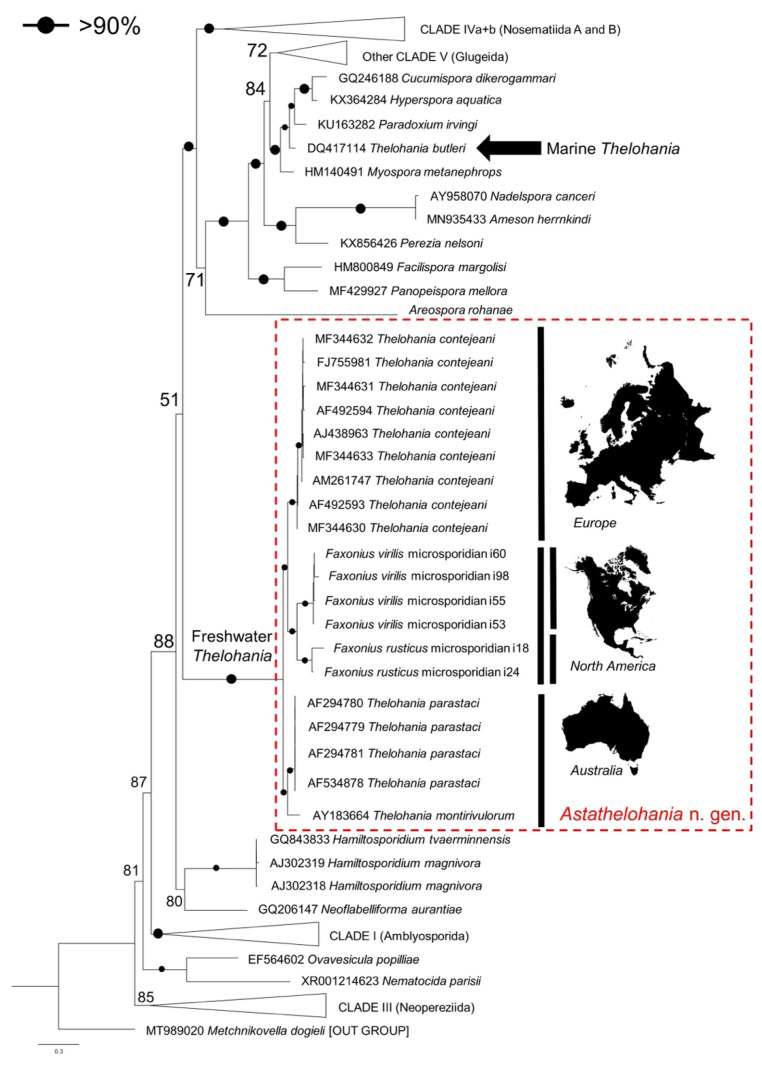
A maximum-likelihood phylogenetic tree of all crayfish-infecting, freshwater *Thelohania* isolates as well as wide-scale Microsporidia representation of each existing clade. The annotated maps demonstrate the distinct crayfish-infecting *Thelohania* species present per continent. The isolates sequenced in this study are denoted on the tree using the host (*Faxonius* sp.) and the microsporidian isolate number. Two isolates are present for a novel microsporidian species from *F. rusticus* (i18 and i24), and four isolates were sequenced from *F. virilis* (i60, i98, i55, i53). The tree was constructed using MAFFT aligned rRNA (SSU) gene sequences followed by IQtree [34]. The tree was annotated in FigTree v.1.4.4.

**Figure 7:**
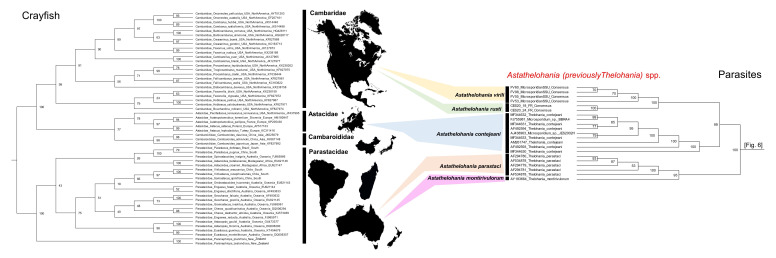
Representative phylogenetic-inferred cladograms of native crayfish species (“Crayfish”) from the families Cambaridae (native geography: North America), Astacidae (native geography: Europe and North America), Cambaroididae (native geography: China and Japan), and Parastacidae (native geography: South America, Madagascar, Australia, New Zealand), compared with microsporidian isolates from the freshwater *Thelohania* (now revised to *Astathelohania*) (“Parasites”). The accession numbers for the isolates are listed by the name of the species on each tree. The microsporidian cladogram was developed from the tree presented in Figure 6. For the “Crayfish” tree, cytochrome oxidase 1 DNA sequence data were aligned using MAFFT and constructed using IQtree [34]. The trees were drawn and annotated in FigTree v.1.4.4.

Phylogenetic analysis revealed that our novel microsporidia grouped in an ‘orphan’ lineage at the base of Clades IV and V, along with the other freshwater *Thelohania* isolates from Europe and Australia (bootstrap: 100%), revealing a genetic similarity between species from specific continental ranges (Figure 6 and Figure 7). Grouping below our microsporidia and the existing freshwater *Thelohania* are the genera *Hamiltosporidium* and *Neoflabelliforma* (Figure 6). The phylogenetic analysis also revealed that freshwater *Thelohania* and marine (‘true’) *Thelohania* spp. are genetically distinct, with *T. butleri* branching separately in Clade V (Figure 6). A sequence demarcation plot of the SSU rRNA gene of all isolates found in the ‘orphan’ lineage, and also comparing *T. butleri*, emphasizes the genetic dissimilarity between freshwater *Thelohania* and marine (‘true’) *Thelohania* with <75% similarity (Figure 5). Therefore, we propose the freshwater members of the genus *Thelohania* be relocated to a new genus, *Astathelohania* gen. et comb. nov., based on genetic and phylogenetic dissimilarity of the 18S rRNA sequences. The novel microsporidia described here are named *Astathelohania virili* n. sp. and *Astathelohania rusti* n. sp., and the species *T. contejeani*, *T. montirivulorum*, and *T. parastaci*, are revised to become members of this genus.

## 4. Taxonomic Summary

### 4.1. Higher Taxonomy

Superphylum: Opisthosporidia (Karpov et al. [36])

Phylum: Rozellomycota (Tedersoo et al. [37]), including the Microsporidia (Balbiani [38]; Wijayawardene et al. [39])

Class: ‘Orphan lineage’ or Clade VI (Dubuffet et al. [29])

Order: Undetermined

Family: Astathelohaniidae Stratton, Reisinger, Behringer, Bojko 2022

Family description: Binucleate, uninucleate, and potentially dimorphic microsporidian parasites that develop within sporophorous vesicles in the muscle tissue of freshwater crustacean hosts. Spores are ellipsoidal, oval, or pear-shaped. Species considered to be members of this family should phylogenetically group with other members of this family using DNA, RNA, or amino acid sequence data, and clade with the type genus and species (*Astathelohania virili*).

Type genus and species: *Astathelohania virili* n. sp. Stratton, Reisinger, Behringer, Bojko 2022

Genus: *Thelohania* (freshwater) replaced by *Astathelohania* Stratton, Reisinger, Behringer, Bojko 2022

*Astathelohania* genus description: This genus should accommodate uninucleate or binucleate species that undergo merogony and sporogony in a sporophorous vesicle. Members of this genus infect freshwater Astacoidea Latreille, 1802 hosts (crayfish), which are globally present. Gene sequence data should be considered when determining the placement of a species into this genus and that data should be used to infer a phylogenetic analysis, showing clustering with other *Astathelohania* species, accounting for possible geographic sequence diversity observed in this study.

Type species: *Astathelohania virili* n. sp. Stratton, Reisinger, Behringer, Bojko 2022

### 4.2. Astathelohania virili n. sp. Stratton, Reisinger, Behringer, Bojko 2022

Species description: The microsporidian parasite infects the muscle and heart tissue of *F. virilis* and undergoes merogony and sporogony in a sporophorous vesicle. The spores are uninucleate and include 16–17 coils of the polar filament. The spores are oval in shape with a wider posterior end and measure 3.4 ± 0.1 µm (SD) in length and 2.0 ± 0.3 µm (SD) in width. To be a candidate for this species, sequence similarity must be shared by comparison to available SSU sequence data for this isolate. Phylogenetically, the parasite must clade with the original sequence provided in this manuscript for *Astathelohania virili*.

Type host: *Faxonius virilis* (Hagen, 1870)

Type locality: South Turtle Lake (46.217698, –89.891143), Vilas County, WI, USA.

Site of infection: This species infects the muscle and heart tissue of the host.

Etymology: The species ‘virili’ is named for the host species (*Faxonius virilis*) in which this novel species was found to infect.

Type material: Histology slides, resin blocks, ethanol-fixed tissue, and glutaraldehyde-fixed tissue are stored at the University of Florida, Reisinger Laboratory. SSU sequence data are deposited in NCBI, under the accession OM630068.

### 4.3. Astathelohania rusti n. sp. Stratton, Reisinger, Behringer, Bojko 2022

Species description: The microsporidian parasite infects the muscle and heart tissue of *F. rusticus* and undergoes merogony and sporogony in a sporophorous vesicle. The spores are uninucleate and include 13–14 coils of the polar filament. The spores are oval in shape with a wider posterior end and measure 3.2 ± 0.5 m (SD) in length and 1.7 ± 0.3 µm (SD) in width. To be a candidate for this species, sequence similarity must be shared by comparison to available SSU sequence data for this isolate. Phylogenetically, the parasite must clade with the original sequence provided in this manuscript for *Astathelohania rusti*.

Type host: *Faxonius rusticus* (Girard, 1852)

Type locality: Darby Creek (40.013388, –83.383180), Madison County, OH, USA.

Site of infection: This species infects the muscle and heart tissue of the host.

Etymology: The species of this parasite ‘rusti’ is named for the host species (*Faxonius rusticus*) in which this novel species was first identified.

Type material: Histology slides, resin blocks, ethanol-fixed tissue, and glutaraldehyde-fixed tissue are stored at the University of Florida, Reisinger Laboratory. SSU sequence data are deposited in NCBI, under the accession OM630067.

### 4.4. Novel and Redescribed Astathelohania Species

*Astathelohania rusti* n. sp. (Stratton, Reisinger, Behringer, Bojko 2022)

*Astathelohania virili* n. sp. (Stratton, Reisinger, Behringer, Bojko 2022)

*Thelohania contejeani* (Henneguy and Thélohan [8]), gen. et comb. nov., *Astathelohania contejeani*

*Thelohania montirivulorum* (Moodie et al. [9]), gen. et comb. nov., *Astathelohania montirivulorum*

*Thelohania parastaci* (Moodie et al. [10]), gen. et comb. nov., *Astathelohania parastaci*

## 5. Discussion

Crayfish can harbor a diverse suite of pathogens, and the freshwater *Thelohania* are a major group of crayfish-infecting microsporidia [4,5]. In this study, we present a taxonomic revision for freshwater *Thelohania* based on SSU rRNA sequence data and phylogenetics, proposing that crayfish-infecting, freshwater members of *Thelohania*, a polyphyletic genus, be transferred to the *Astathelohania* gen. et comb. nov., housed in the family Astahelohaniidae n. fam., making a clear distinction from the Clade V family, Thelohaniidae, which now houses marine and terrestrial *Thelohania* spp. In addition, we describe two new species of *Astathelohania*, *Astathelohania virili* n. sp. and *Astathelohania rusti* n. sp., from two crayfish hosts in North America, using histopathology, ultrastructure, intracellular development, and SSU phylogenetics.

### 5.1. Renaming the Freshwater Thelohania to Astathelohania n. gen.

As genetic data become increasingly available for microsporidia, it has become clearer that traditional data (e.g., phenotypic, ecological, developmental) alone are unable to delineate accurate phylogenies—a combination of these data are required to currently identify species and their taxonomy, evident by several recent species revisions [40,41]. For some of the first microsporidian genera described, such as the *Nosema*, it has proven vital to incorporate genetic data as part of a revision [41]. Several studies have called for a taxonomic revision of the polyphyletic genus *Thelohania* since it has become increasingly apparent that the marine *Thelohania* and the freshwater *Thelohania* are not closely related genetically and are in fact clades apart [7,15,21]. Other studies have begun to revise the polyphyletic genus by placing terrestrial *Thelohania* species into more appropriate genera based on genetic, phylogenetic, developmental, and ecological data [23,24]. Our study provides further evidence to support taxonomic revision through the discovery of two new species in this ‘orphan lineage’.

Based on our phylogenetic analysis, freshwater *Thelohania* branch outside of both Clades IV and V, and importantly branch together in a well-supported group separate from the marine *T. butleri* (Clade V), the only ‘true’ *Thelohania* species with genetic data available (Figure 6). Our taxonomic revision is further supported by several recent studies [15,29]. The sequence demarcation plot we provide illustrates the dissimilarity between marine and freshwater *Thelohania*, based on the SSU rRNA gene (Figure 5).

Further, the family Thelohaniidae remains polyphyletic and also requires taxonomic revision [42]. Many of the genera and species assigned to this family have undergone recent revision on the basis of genetic dissimilarity [23,24,43,44,45]. Our phylogenetic tree further highlights the need for the novel *Astathelohania* genus to be placed into a new family (Astathelohaniidae n. fam.) considering that all crayfish-infecting, freshwater *Thelohania* do not fall into the same clade as any genetically validated members of the family Thelohaniidae (Figure 6) [39].

Therefore, we propose a revision in which the crayfish-infecting, freshwater members of the *Thelohania* are distinguished and relocated to the *Astathelohania* n. gen. and Astathelohaniidae n. fam. This new genus and family are named for the *Thelohania*, maintaining their important historic connotations, but additionally represent the freshwater crayfish hosts of this genetically distinct lineage, helping to maintain the historic genus and family names that once represented these species for over a century of published literature.

### 5.2. Two Novel Crayfish Parasites in the USA

To date, seven microsporidia have been formally described from crayfish hosts, but none of these are known from the crayfish genus *Faxonius* [5]. The genus *Faxonius* is the third most species-rich genus of crayfish in the world behind *Procambarus* and *Cambarus*, yet little is known about the pathogens this group harbors [4,46]. *Astathelohania virili* n. sp. and *A. rusti* n. sp. are the first formally described microsporidia found to infect members of the genus *Faxonius*. Both crayfish hosts, *F. virilis* and *F. rusticus*, have invasive ranges throughout North America, but these novel parasites were found in the native range of each host. Further research should examine whether these novel parasites are found in the invaded ranges of the crayfish hosts.

There have been reports of two suspected *T. contejeani* infection within North America and an unofficial *T. cambari* species reported [17,18,19]. These reports were all based on the observation of octosporous development and spore size. However, the size range of spores for the suspected *T. contejeani* infections overlap with both our spore size ranges and the range described for *A. contejeani* and *A. parastaci* (Table 2) [10,15,17,18]. We also now know of many microsporidian groups that undergo octosporous development within an SPV outside of *Thelohania* [15,47]. Therefore, until these infections are rediscovered, and genetic data become available, we cannot say whether these reports are accurate. Similarly, the unofficial species *T. cambari* was placed in the genus based on spore size and observation of octosporous development [19]. The spores were much larger in size than our *Astathelohania* species but do overlap with the size range reported for binucleate spores of *A. montirivulorum* and *A. parastaci* (Table 2) [9,10,19]. Genetic and ultrastructural data must become available before *T. cambari* can be formally recognized.

### 5.3. Host–Parasite Co-Evolution

The discovery of these novel parasites allowed us to examine the possibility of host–parasite co-evolution of crayfish hosts and *Astathelohania* microsporidia. Phylogenetic studies of the superfamily Astacoidea illustrate that the divergence of families and genera are geographically affiliated [46,48]. Families in the Northern (Cambaroididae, Astacidae, and Cambaridae) and Southern (Parastacidae) hemispheres diverged over 265 mya [49]. The family Cambaridae is the youngest yet most diverse crayfish lineage, undergoing diversification and radiation approximately 90 mya [50].

The diversity observed within the *Astathelohania* genus may also represent a geographic split. The microsporidia *A. montirivulorum* and *A. parastaci* are only known from the Australian crayfish *C. destructor* in the family Parastacidae [9,10]. *Astathelohania contejeani* has been found to infect three members of the family Astacidae which include *A. pallipes*, *Astacus astacus*, and *P. leniusculus*, and all isolates were discovered in Europe [13,14,15]. Finally, *A. virili* and *A. rusti* infect two members of the North American family Cambaridae. Our sequence demarcation plot highlights that isolates discovered in the oldest host family (Parastacidae) are least similar to isolates from the youngest family (Cambaridae) (Figure 5, Figure 6 and Figure 7). There is little genetic variation between European isolates since they are all the same microsporidian species; however, two strains of *A. contejeani* have been described and are evident in the phylogenetic tree (Figure 5 and Figure 6) [13]. In Australia, the *Astathelohania* (*A. montirivulorum* and *A. parastaci*) infect the same host species and are 93% similar to one another [9,10]. In North America, *A. virili* and *A. rusti* show considerable genetic variation which may be because North American crayfishes are a significantly more diverse group compared to crayfishes in the families Astacidae and Parastacidae. Therefore, if there is a host–parasite co-evolution it would make sense that their parasites would also be more genetically diverse.

## 6. Conclusions

It is a vital taxonomic step to separate the crayfish-infecting, freshwater *Thelohania* into their own distinct genus, avoiding polyphyly in ongoing taxonomic studies concerning the ‘true’ marine *Thelohania*. Here, we have provided a description of the *Astathelohania* n. gen., in the family Astathelohaniidae n. fam., to provide valuable systematic distinction for this lineage. This has resulted in three species of *Thelohania* being revised and the addition of two new species. The two new species we describe provide a North American perspective of *Astathelohania* diversity, which is now viewed as a globally diverse genus. We see well-supported groups in our phylogeny, which combine all suggested *Astathelohania* species with 100% bootstrap support, as well as splitting the various genera based on geography and host diversity.

## Figures and Tables

**Table 1:** Sampling detail of each individual crayfish collected with indication of what microsporidian species each crayfish was infected with and the data available for each crayfish.

Host Species	Site	Coordinates	Collection Date	Sex	Carapace Length (mm)	Microsporidian Species	SSU	Histology	Electron Microscopy	Accession Number
*F. rusticus*	Darby Creek, OH	40.013388, −83.383180	30 June 2021	MII	27	*A. rusti* n. sp.	✓	✓	—	OM630066
*F. rusticus*	Darby Creek, OH	40.013388, −83.383180	30 June 2021	MI	32	*A. rusti* n. sp.	✓	✓	✓	OM630067
*F. virilis*	South Turtle Lake, WI	46.217698, −89.891143	09 July 2019	MII	51	*A. virili* n. sp.	✓	✓	✓	OM630068
*F. virilis*	South Turtle Lake, WI	46.217698, −89.891143	09 July 2019	MII	50	*A. virili* n. sp.	✓	✓	—	OM630069
*F. virilis*	South Turtle Lake, WI	46.217698, −89.891143	09 July 2019	MII	43	*A. virili* n. sp.	✓	✓	—	OM630070
*F. virilis*	Crab Lake, WI	46.203368, −89.729255	19 July 2019	MII	40	*A. rusti* n. sp.	✓	✓	—	OM630071

**Table 2:** Comparison of morphological features of all described *Astathelohania* (previously *Thelohania*) species. In addition, the morphological features of two suspected and an unofficial *Thelohania* in North America are included. The table was adapted from Moodie et al. [9], n/a indicates data were not available.

Morphological feature	*A. rusti* n. sp.		*A. virili* n. sp.		*A. montirivulorum*	*A. parastaci*	*A. contejeani*	*A. contejeani*		“*T. contejeani*”		“*T. contejeani*”	“*T. cambari*”
					Moodie et al. [9]	Moodie et al. [10]	Lom et al. [13]	Pretto et al. [15]		Graham and France [17]		McGriff and Modin [18]	Sprague [19]
Shore shape	Oval, wider posterior end		Oval, wider posterior end		Lozenge, round ends	Lozenge, round ends	Oval, wider posterior end	Oval, wider posterior end		Oval		Oval	Oval, wider posterior end
Uninucleate spore length (μm)	3.2 ± 0.5 ^1^	*n* = 10	3.4 ± 0.1 ^1^	*n* = 7	n/a	n/a	4.2 ^2^	3.6 ± 0.4 ^2^	*n* = 50	3.3 (2.8–3.6)	*n* = 50	3.0–3.8	4.6
Uninucleate spore width (μm)	1.7 ± 0.3^1^	*n* = 10	2.0 ± 0.3 ^1^	*n* = 10	n/a	n/a	2.1 ^2^	2.3 ± 0.3 ^2^	*n* = 50	2.2 (2.0–2.6)	*n* = 50	1.8–2.4	2.2
Binucleate spore length (μm)	n/a		n/a		5.9 (4.9–7.2) ^2^	3.9 (3.2–4.9) ^2^	3.8 ^2^	3.3 ± 0.5 ^2^	*n* = 50	n/a		n/a	n/a
Binucleate spore width (μm)	n/a		n/a		2.6 (2.0–3.1) ^2^	2.0 (1.5–2.7) ^2^	1.8 ^2^	1.7 ± 0.2 ^2^	*n* = 50	n/a		n/a	n/a
Uninucleate—no. coils in polar filament	13–14		16–17		20–22	11–20	9–10	9–12		n/a		n/a	n/a
Uninucleate—polar filament diameter (nm)	141 ± 14	*n* = 10	118 ± 3	*n* = 10	98 (82–111)	59 (53–74)	120–180 ^3^	77	*n* = 10	n/a		n/a	n/a
Binucleate—no. coils in polar filament	n/a		n/a		20–22	6–8	5–7	5–6		n/a		n/a	n/a
Binucleate—polar filament diameter (nm)	n/a		n/a		107 (90–140)	83 (65–102)	n/a	108	*n* = 10	n/a		n/a	n/a
SPV diameter (μm)	5.2 ± 0.6 ^1^	*n* = 10	8.1 ± 0.7 ^1^	*n* = 10	8.4 (7.0–9.6) ^2^	8.8 (7.4–10.5) ^2^	8–9 ^3^	9.4 ± 0.6 ^2^	*n* = 20	7.9 (6.4–8.1)	*n* = 10	n/a	n/a
SPV tubular-like structure diameter (nm)	244 ± 32	*n* = 10	241 ± 26	*n* = 10	171 (130–249)	249 (205–307)	220	155–185	*n* = 20	n/a		n/a	n/a
SPV microtubular-like structure diameter (nm)	70 ± 9	*n* = 10	73 ± 10	*n* = 10	85 (63–117)	73 (50–99)	80–100	75–85	*n* = 20	n/a		n/a	n/a
Lateral exospore thickness of uninucleate spores (nm)	25 ± 6	*n* = 10	25 ± 3	*n* = 10	31 (30–40)	24 (20–40)	24–30 ^4^	28	*n* = 15	n/a		n/a	n/a
Lateral endospore thickness of uninucleate spores (nm)	57 ± 18	*n* = 10	82 ± 12	*n* = 10	108 (80–130)	73 (56–110)	60–90 ^4^	78	*n* = 15	n/a		n/a	n/a
Lateral exospore thickness of binucleate spores (nm)	n/a		n/a		22 (17–30)	34 (30–40)	n/a	32	*n* = 8	n/a		n/a	n/a
Lateral endospore thickness of binucleate spores (nm)	n/a		n/a		65 (40–80)	58 (50–60)	n/a	55	*n* = 8	n/a		n/a	n/a
Dimorphic sporogony?	No		No		Yes	Yes	Yes	Yes		n/a		n/a	n/a

^1^ Resin infiltrated. ^2^ Light microscopy. ^3^ Cossins and Bowler [11]. ^4^ Vivares [12].

## Data Availability

Histology slides, resin blocks, ethanol-fixed tissue, and glutaraldehyde-fixed tissue are stored at the University of Florida, Reisinger Laboratory. SSU sequence data are deposited in NCBI, under the accession numbers OM630066–OM630071.
